# iFO (infrared Fish Observation) – An open source low-cost infrared underwater video system

**DOI:** 10.1016/j.ohx.2020.e00149

**Published:** 2020-10-08

**Authors:** Andreas Hermann, Jérôme Chladek, Daniel Stepputtis

**Affiliations:** Thuenen Institute of Baltic Sea Fisheries, Alter Hafen Süd 2, Germany

**Keywords:** Open source, Infrared camera, Underwater video, Fish, Marine life, Observation, Animal behaviour

## Abstract

Underwater video surveillance is an important data source in marine science, e.g. for behaviour studies. Scientists commonly use water resistant ruggedized monitoring equipment, which is cost-intensive and usually limited to visible light. This has two disadvantages: the observation is limited to space and time where visible light is available or, under artificial illumination, behaviour of marine life is potentially biased. Infrared (IR) video surveillance have been used before to overcome these. It records videos at visible light and under IR-illumination. With today’s efficiency of IR-LED and video technology even low-cost systems reach visibility ranges suited for many application scenarios. We describe a low-cost open-source based hardware/software system (iFO). It consists of a single-board computer controlling the camera and lamps (with high power IR-LEDs), printed circuit boards (PCB), the underwater housings and 3D-printable models to mount PCBs in the housings and the housings to standard GoPro mounts. The Linux based software includes webserver, remote control, motion detection, scheduler, video transfer, storage at external hard disk and more. A ready-to-use SD-card image is included. We use rugged underwater housings with 100 m (optional 400 m) depth ratings. Finally, we describe a typical application observing the behaviour of cod in fish pots.

## Graphical abstract

**Figure:**
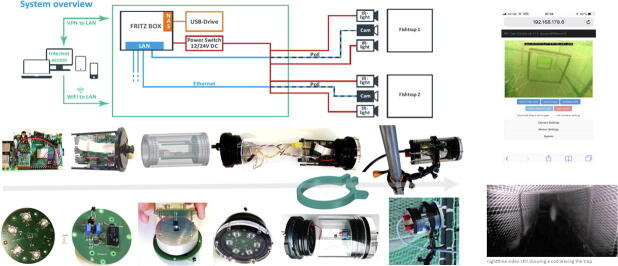

## Specifications table

**Table:** 

Hardware name	*iFO- infrared Fish Observation*

Subject area	• Biological Sciences (Marine and Fishery research) • Educational Tools and Open Source Alternatives to Existing Infrastructure
Hardware type	• Underwater imaging tools • Field measurements and sensors • Electrical engineering and computer science
Open source license	*Software: MIT* *Hardware: SHL-2.0*
Cost of hardware	*1 × camera = 220 € (100 m depth rated housing),* *2 × infrared lamps = 2 × 155 € = 310 € (100 m depth rated housing)*
Source file repository	https://doi.org/10.17605/osf.io/9xuz2
Project repository	https://doi.org/10.17605/osf.io/tckpg

## 1 Hardware in context

We present “infrared Fish Observation” (iFO), an open-source low-cost underwater infrared (IR) video observation system using high power LEDs and low-cost CMOS camera modules. The use of IR-video surveillance at night is very common for onshore applications and therefore hardware became very efficient at low-cost. On the other hand, in marine science, the use of visible light in dark environments is mostly inappropriate to avoid bias of fish behavior. Acoustic cameras have been used for this purpose [7], but those are complex and expensive systems with low resolution compared to optical cameras. Like human eyes, many marine species, such as fish, cannot see IR-light. One major obstacle is the relative high attenuation of IR-light under water compared to visible light [2], [3], [4]. That limits this method to short-range observations. Nevertheless, IR-video surveillance have been used for underwater observations in dark environments earlier, e.g. [1], [5], [6], [8], [9]. These studies do not give detailed description of the IR systems and therefore are not reusable. Additionally, since the time of their development, technology has made great improvements. With today’s effectivity of LED technology, even low-cost CMOS cameras can cover ranges of observation >2 m, suited for many application scenarios. Furthermore, single board computers like Raspberry Pi or BeagleBone are inexpensive and can be operated with freely available open source operating systems (Linux based). They are used in many different application areas and a wide range of open-source software tools are free available. This makes them ideal for quickly realizable developments of highly adaptable cost-effective systems.

## 2 Hardware description

As there were no affordable underwater IR systems available, we developed the open-source system iFO (infrared Fish Observation). iFO uses a consumer single-board computer (Raspberry Pi) and standard industry parts. The Raspberry Pi single-board computers have been applied for marine supervision and fish observation earlier, e.g. [20], [21], [22].

In our application one system consists of one camera and two lights, whereby components cost around 530€ including 100 m depth rated housings. With the reuse of existing open-source software and hardware, that were adapted to our scientific needs, we achieved a sustainable ocean monitoring system at low-cost. The system offers a webserver, a comfortable scheduler, a motion detection unit and can store internally more than one week continuous video data.

We present iFO in a typical application where we observe the behaviour of cod (Gadus morhua, L. 1758) at the entrance of different fish pots with the aim to improve the catch effectivity. It delivers 24/7 underwater video footage in a range up to 2 m at infrared illumination and much greater distances at daylight. Additionally, we use a LTE router (FritzBox 6890) with a 2 GB swappable hard disk to be used with up to four camera systems. This allows video data storage for several weeks and provides full access via VPN and LTE to the whole system in the field. It gives remotely live videos, access to the cameras’ webserver for adjustment and setup, for instant download of data and to the cameras’ operating system for maintenance.

### 2.1 Spectral sensitivity of the camera

**Fig. 1:**
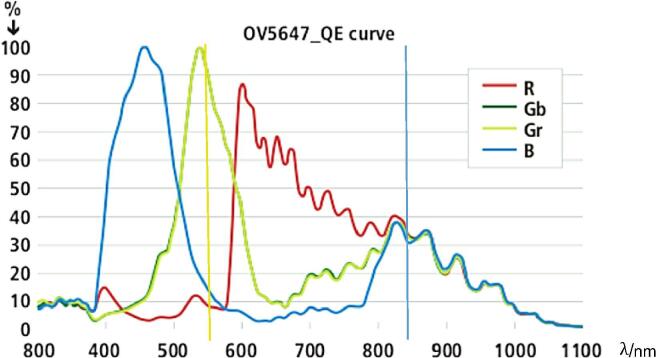
Spectral quantum efficiency of the CMOS camera [14]. The sensitivity at the medium wavelength of the IR lamp (850 nm) is marked with a blue line and the sensitivity at the wavelength with the highest sensitivity of human eyes (555 nm) is marked with a yellow line. (For interpretation of the references to colour in this figure legend, the reader is referred to the web version of this article.)

The CMOS camera has an OV5647 [13] sensor chip, which has no IR filter. We use lenses that do not have IR filter either. As typical for colour camera sensors, the photoactive area is evenly distributed in red, green and blue filtered pixels. Its relative spectral quantum efficiency is shown in Fig. 1. Quantum efficiency (QE) is measured over a range of different wavelengths to characterize a device's efficiency at each photon energy level. It shows the fraction of emitted electrons *e* from a given number of photons illuminating the sensor. That means, e.g. a number of 100 photons of 555 nm wavelength generates 90 electrons (yellow line) on a green filtered pixel and a number of 100 photons of 850 nm wavelength generates 35 electrons (blue line) on the same pixel.

Each colour (blue, green and red) represents a third of the full active sensor area. Therefore, the total quantum efficiency of the active area for a monochromatic light at a certain wavelength can be calculated from the sum of the electrons on each coloured pixel type, e.g. for 555 nm it is the sum of 15e at blue, 90e at green and 8e at red pixel equals to 15e + 90e + 8e = 113e. It can be seen that the quantum efficiency for infrared light at 850 nm (@850 nm: 35e + 35e + 35e = 105e) is very similar to those in the visible range (@555 nm: 15e + 90e + 8e = 113e). Consequently, the camera can be used for visible light and IR-light.

The datasheet also specifies a photometric sensitivity of $\frac{680mV}{\mathrm{lx}∙s}$ and a dark current value of *16 mV/s* [13] (see Table 1).

**Table 1:** components of iFO IR underwater observation system.

Part	Component	Type	Source	chapter
IR-server camera	Enclosure	Mechanical (from supplier)	external supplier	3.1, 3.3.1
	Single board computer	Printed circuit board	external supplier	3.3.2
	IR camera board	Printed circuit board	external supplier	3.3.3
	Real time clock	Printed circuit board	external supplier	3.3.4
	PCB IR-serverV11	Printed circuit board	Sourcefile (Eagle)	3.3.5
	Mount for PCB	Mechanical (3D printable)	Sourcefile (STL)	3.3.6
	Mount for GoPro	Mechanical (3D printable)	Sourcefile (STL)	3.2
IR-lamp	Enclosure	Mechanical (from supplier)	external supplier	3.1, 3.4.1
	PCB IR-LampV31	Printed circuit board	Sourcefile (Eagle)	3.4.2
	PCB IR-LampV32	Printed circuit board	Sourcefile (Eagle)	3.4.2
	Aluminium heat drain	Mechanical	Sourcefile (STL)	3.4.3
	Mount for GoPro	Mechanical (3D printable)	Sourcefile (STL)	3.2

### 2.2 Spectrum and intensity of the IR lamps

**Fig. 2:**
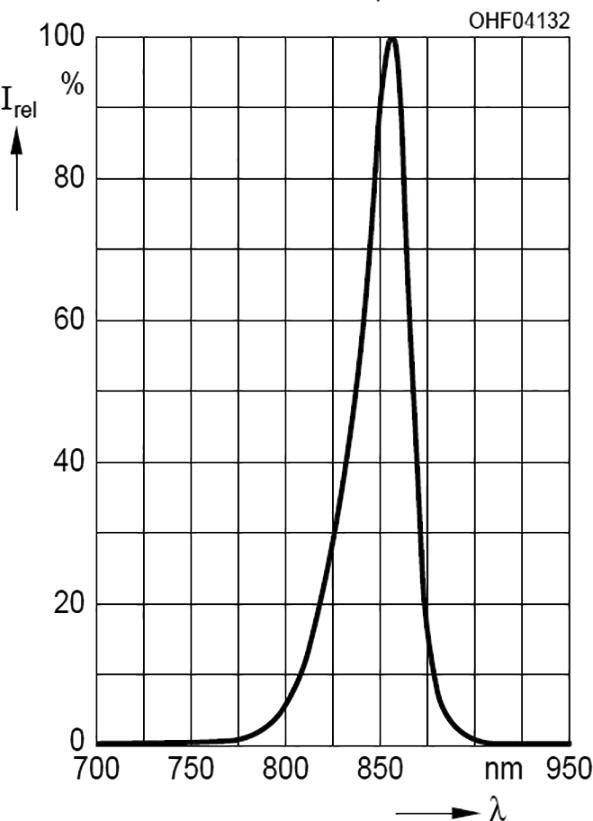
Relative spectral intensity of the LED lamp.

Each infrared lamp uses six Osram SFH4715AS IR-LED’s [15] with a beam angle of 90°. The beam angles can be adapted by optical lenses mounted on the PCB (Table 11: no. 15, 15a). The total electrical power is $P_{el}=n∙U∙I=6∙3.15V∙1A=18.9W$. The centroid wavelength is 850 nm with a radiant power $\phi_{e}=1.530W$per LED, resulting in $\phi_{e}=$9.180 W per lamp. Fig. 2 shows the relative spectral intensity.

## 3 Hardware components

### 3.1 Enclosure

The housings for the IR-server camera and the IR-lamp are taken from the Blue Robotics Inc. 3″-series. Technical details and 3D-drawings can be downloaded at [16]. We use the acrylic tube version that is depth rated to 100 m, an aluminium version rated to 400 m is also available. The end caps have through-holes for cable feed-through penetrators, whereas different types of penetrators are available [17]. The cable feed-through is sealed with marine epoxy to build a safe custom made cable confection. A detailed tutorial is given at [18].

### 3.2 GoPro mount for IR-lamp and IR-server

**Fig. 3:**
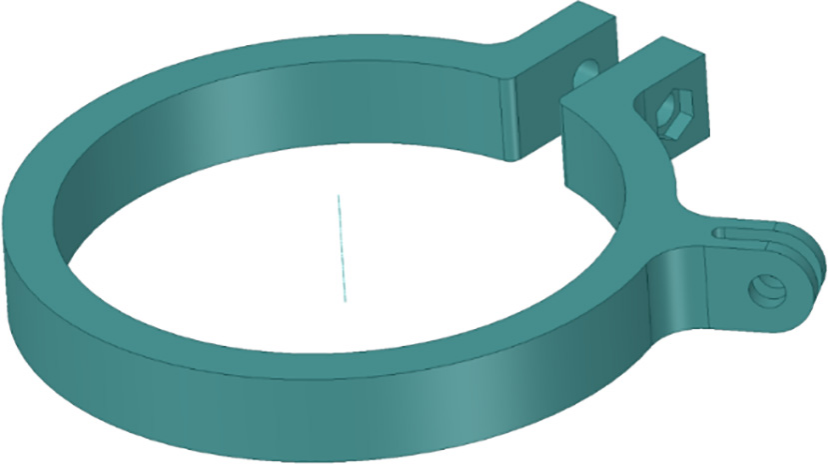
3D-printable mounting device for IR-lamp and IR-camera for GoPro mounts.

The GoPro mount (see Fig. 3 fits to the outer surface of the acrylic tube. It is tightened by a M5 × 35 mm screw (hexagon socket ISO10642) with M5 hexagon nut. We have used ABS material for 3D-printing to improve stability of the GoPro-mount under water and cold conditions. The use of standard PLA was insufficient because it became brittle in saltwater after a few weeks.

### 3.3 Server/Camera

In the server/camera enclosure, four printed cicuit boards (PCB) are connected to each other and tied on a 3D printable mount.

1 Raspberry Pi single-board computer [12]
2 PCB with Real time clock
3 PCB with the camera module
4 PCB ‘IR-server’ with voltage regulator and two output drivers.

#### 3.3.1 Enclosure Server/Camera

For the server/camera a 298 mm acrylic tube described in chapter 3.1 is sectioned in two halfes to get enclosures for two server/cameras. For a set of two cameras the following parts (see Table 2), are needed:

**Table 2:** Bill of material for a set of two IR-server/camera housings.

Item	No.	Description	Manufacturer Code	Price/€
1	1	Cast Acrylic Tube – 11.75″, 298 mm (3″)	WTE3-P-TUBE-12-R1-RP	46.00
2	4	O-Ring Flange (3“)	WTE3-M-FLANGE-SEAL-R2-RP	96.00
3	2	Aluminium End Cap (3″)	WTE3-M-END-CAP-R1-RP	20.00
4	2	Clear Acrylic End Cap (3″)	WTE3-P-END-CAP-R1-RP	20.00
5	2	Enclosure Vent and Plug	VENT-ASM-R1-RP	16.00
6	4	M10 Cable Penetrator for 8 mm Cable	PENETRATOR-M-BOLT-8MM-10-25-R2-RP	20.00
Total				218.00
Total for one piece				109.00

Table 3 shows a distributor list for the enclosure. For sealing the cable penetrator we use potting compound (Table 8, Item15). The 100 g package can be used for about 35 cable penetrators, when all are sealed with one compound mix (within ½ an hour).

**Table 3:** Distributor list for IR-server camera housings.

1:	www.bluerobotics.com	2:	www.nido.ai	3:	www.igp.de

#### 3.3.2 Raspberry Pi single-board computer

**Fig. 4:**
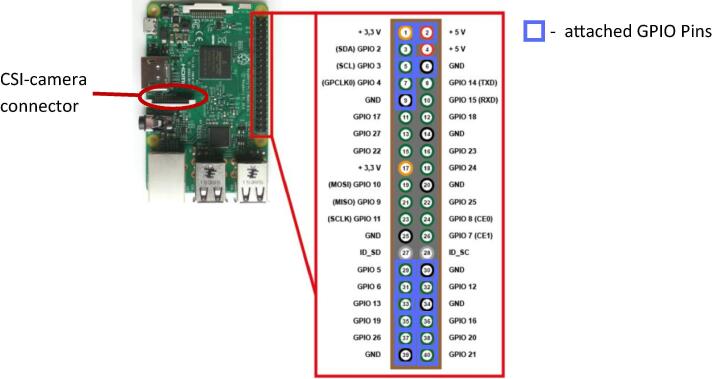
RaspberryPi interfaces in use: CSI-camera connector and GPIO connector with attached pins.

The Raspberry Pi single-board computer has a CSI interface for the camera board and a 40 pin-GPIO interface used here for the PCB RTC and the PCB ‘IR-server’ with power converter and two output drivers (see Fig. 4).

The assembly of the PCB ‘IR-server’ is documented in chapter 3.3.7. The Raspberry Pi with additional PCBs and the camera PCB is fixed to a 3D-printable mount that fits into the underwater housing. The mechanical details of this mount are specified in chapter 3.3.6. The bill of material is shown in Table 4.

**Table 4:** Bill of material for the Raspberry Pi.

Item	No.	Description	Distributor / Code	Manufacturer / Code	Price/€
1	2	Raspberry Pi 3 Model B+	de.rs-online.com 137–3331	Raspberry Pi 3 Model B+	58.94
2	2	Micro SD Karte, MicroSDXC 64 GB, Class 10, UHS-I U3	de.rs-online.com 174–4627	Kingston SDCR/64 GB	45.98
Total					104.92
Total for one piece					52.46

#### 3.3.3 PCB IR-camera

The camera PCB uses the 5MP OV5647 CMOS sensor IC, which has no IR-filter. It is supplied with CSI interface and cable. The M12 S-mounted lenses are without IR-filter, either, different angles of sight are available.The bill of material is shown in Table 5.

**Table 5:** Bill of material for IR camera PCBs.

Item	No.	Description	Distributor / Code	Manufacturer / Code	Price/€
1	2	RPi IR camera (F) (75° lens)	www.exp-tech.de EXP-R63-017	Waveshare SKU: 10299 Part:: Rpi Camera (F) UPC: 700646949915	77.20
1a	2	RPi IR camera (H) (fisheye lens)	www.exp-tech.de EXP-R63-019	Waveshare SKU: 10703 Part:: Rpi Camera (H) UPC: 799632838333	119.56
Total for one piece (75° lens)					38.60

#### 3.3.4 PCB Real time clock

Various RTC modules are available for the Raspberry Pi. The most appropriate one is from SERTRONICS as it only uses five pins of the GPIO header at a low price. The bill of material is shown in Table 6.

**Table 6:** Bill of material for the real time clock.

Item	No.	Description	Distributor / Code	Manufacturer / Code	Price/€
1	2	Real Time Clock for Raspberry Pi	www.reichelt.de RPI RTC CLOCK	SERTRONICS RPI-RTC	5.50
Total for one piece					2.75

#### 3.3.5 PCB ‘IR-server’

**Fig. 5:**
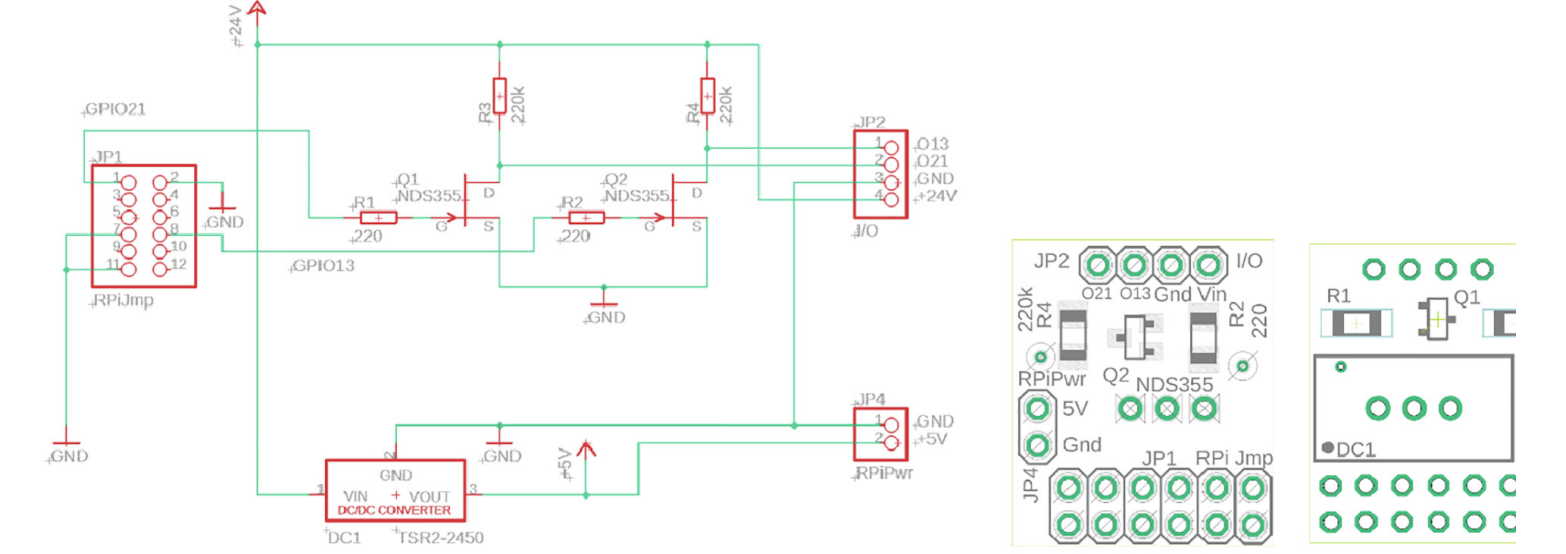
PCB ‘IR server’: left: Schematic view; right: PCB top and bottom view.

The PCB ‘IR-server’ is mounted via the 2 by 6-pin-socket JP1 to the GPIO pin-header of the Raspberry Pi Table 7. On the topside of the PCB, there are two pin-headers JP2 and JP4. The 4-pin I/O header JP2 is connected to the 24VDC input power and the two output drivers (O13 out and O21 out) that allow a remote control of two independent groups of lamps. In our standard applications we control the lamps by light sensors. For remote controlled lamps some modifications are necessary, which are described in chapter 3.4.5. For the standard setup, the 2-pin header JP4 delivers the 5VDC@2A power for the Raspberry Pi. It is connected via a 2-wire line with pin-sockets to the power pins of the Raspberry Pi. Fig. 5 shows the schematic and the top and bottom view of the PCB ‘IR server’. Table 7 the jumper connections and Table 8 the bill of material.

**Table 7:** PCB ‘IR server’ jumper connections.

JP1					
Pin	to RPI Pin	function	Pin	to RPI Pin	function
1	40	GPIO21, Lamp2	8	33	GPIO13, Lamp1
2	39	GND	9…10	32…31	n.c.
3…6	38…35	n.c.	11	30	GND
7	34	GND	12	29	n.c.

**JP2**		
**JP2-Pin**	**to cable**	**function**
1	Lamp control 1	O13out
2	Lamp control 2	O21out
3	GND	GND
4	+24VDC in	+24VDC in
**JP4**		
**JP4-Pin**	**to RPI Pin**	**function**
1	6	GND
2	4	+5V DC

**Table 8:** Bill of material for PCB ‘IR-server’

Item	No.	Description	Distributor / Code	Manufacturer / Code	Price/€
1	2	Resistor SMD-1206 220kOhm 5% 0.5 W	de.rs-online.com 807–1173	TE-Connectivity CRGH1206J220K	0.058
2	2	Resisitor SMD-1206 220 Ohm 5% 0,5W	de.rs-online.com 807–1176	TE-Connectivity CRGH1206J220R	0.056
3	2	Transistor NDS355AN SOT23	de.rs-online.com 739–0167	Fairchild NDS355AN	0.54
4	1	DC/DC Converter Traco TSR2-2450 SIP3	de.rs-online.com 166–6063	Tracopower TSR2-2450	10.14
5	4/90	Pin socket 2 × 6 2.54 mm	de.rs-online.com 230–4922	Stelvio Kontek 90x2-pin 613,090,271,123	0.23
6	4/36	Pin socket 1 × 4 2.54 mm	de.rs-online.com 549–0026	E-TEC 36-pin-socket BL1-036-G-700–01	0.37
7	2/36	Pin socket 1 × 2 2.54 mm	de.rs-online.com 549–0026	E-TEC 36-pin-socket BL1-036-G-700–01	0.19
8	4/20	Pin header 1 × 4 2.54 mm	de.rs-online.com 360–6342	Molex C-Grid III 20pin 90210–0780	0.40
9	2/20	Pin header 1 x2 2.54 mm	de.rs-online.com 360–6364	Molex C-Grid III 20pin90° 90121–0780	0.18
10	4/100	Slotted screw M2 × 12 mm	de.rs-online.com 560–710	RSPRO, steel galvanized DIN84-M2x12	0.21
11	4/250	Hexagon nut M2 × 4 mm	de.rs-online.com 560–271	RSPRO, steel galvanized	0.09
12	4/100	Spacer round, Ø 6 × 3 mm, 3.2 mm drill	de.rs-online.com 102–6110	Richco 469.09.03	0.48
13	4/500	Tapping screw 2.2 × 6 mm	online-schrauben.de DIN7971-C-2,2X6,5	Steel galvanized DIN7971-C-2,2X6,5	0.24
14	4/500	Slotted screw M3 × 8 mm	online-schrauben.de DIN84-4.8-M3X8	Steel galvanized DIN84-M3x8	0.08
15	3/100	Potting compound 100 g	de.rs-online 199–1418	RS PRO Epoxid 2x50g	0.27
16	1/12	PCB IRserver_V11	www.aisler.net	PCB for playground 12pcs	1.21
Total for one piece					14.74

#### 3.3.6 PCB mount (3D-printable)

**Fig. 6:**
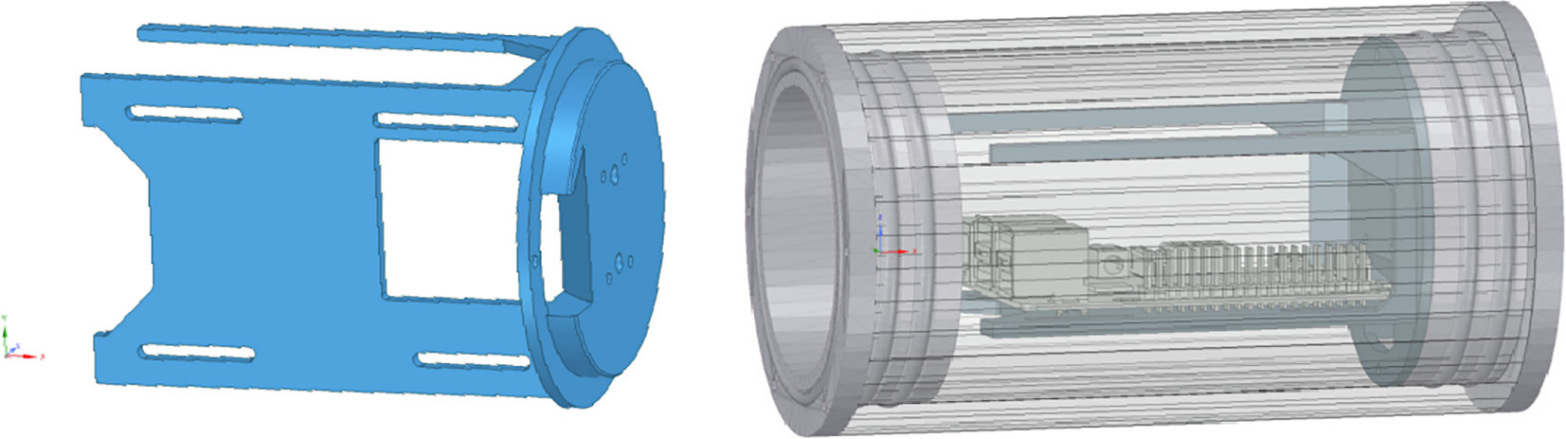
Drawings of the 3D-printable mount inside the IR-server enclosure; left: PCB mount, right: schematic view of Raspberry Pi, mounted on the PCB mount inside the enclosure.

Fig. 6 shows the 3D-printable mount that holds the Raspberry Pi with its additional PCB boards in the acrylic tube and the CMOS camera at the centre in front of the acrylic window. It is attached to the flange with four screws.

#### 3.3.7 Assembly

**Fig. 7:**
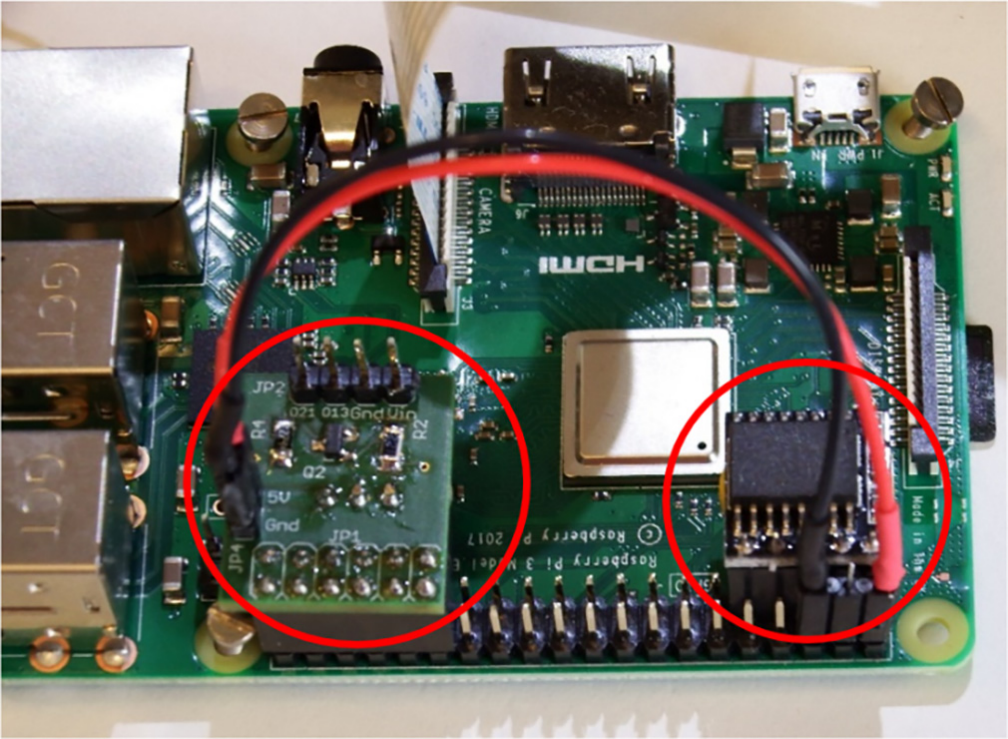
PCB ‘IR-server’ and PCB RTC connected to the Raspberry Pi’s GPIO-header.

For the assembly of the server/camera, the PCB of the RTC and the ‘IR-server’ are mounted to the GPIO header of the Raspberry Pi. The PCB ‘IR-server’ is connected to the Raspberry Pi GPIO-pins #29 to #40. The PCB of the RTC to the odd pin row from #1 to #9. The power supply for the Raspberry Pi is connected by a two wire cable with pin sockets between JP4 at PCB ‘IR-server’ and Raspberry Pi GPIO-pins #2 (+5V) and #6 (GND). All connections are marked in red in Fig. 7.

**Fig. 8:**
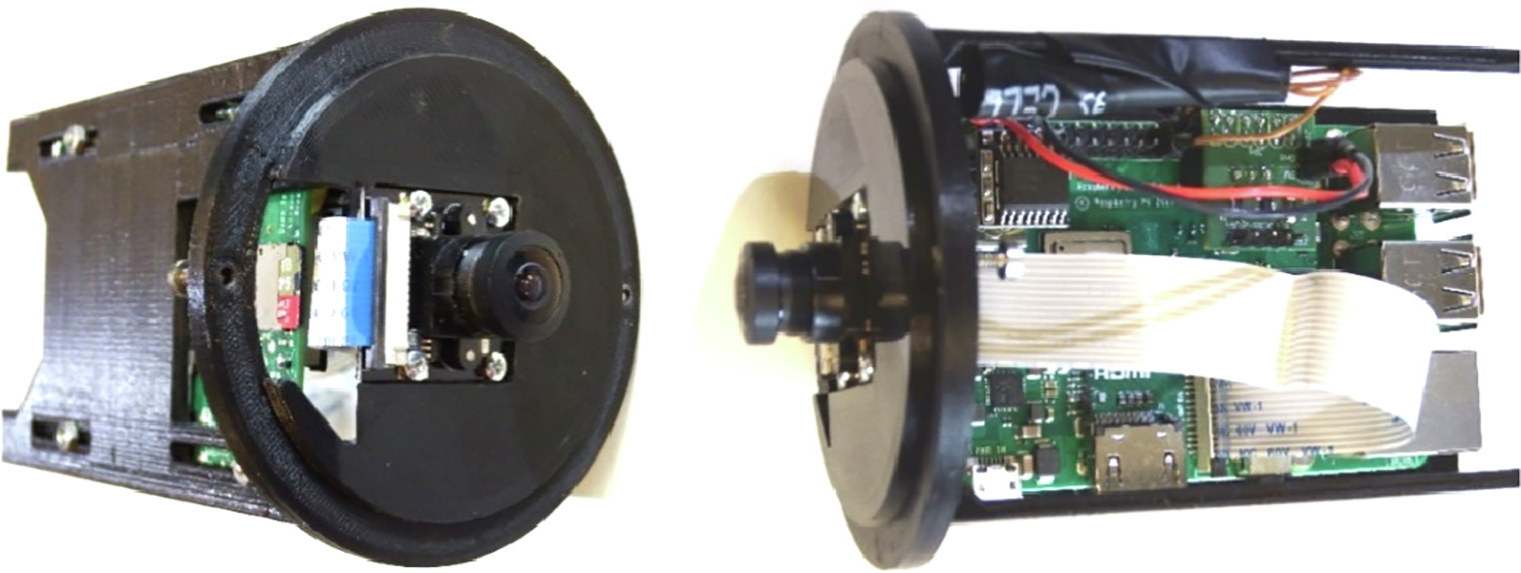
Attaching the camera and the Raspberry Pi PCBs to the 3D-printed PCB mount.

We use Raspberry Pi 3 Model B + but we also tested older versions (Pi 3 Model B and Pi 2 Model B). The camera module is without infrared filter. Different types of optics are available e.g. 160° or 75° degrees angle of view. The operating system with all necessary modules, setups and software components is written to the SD memory card. It is recommended to use 64 GB (or greater) SDXC memory card, to have enough space to locally store videos. The Raspberry Pi with the attached PCBs and the camera module are mounted to the 3D-printed mount that fits to the flange (Fig. 8).

**Fig. 9:**
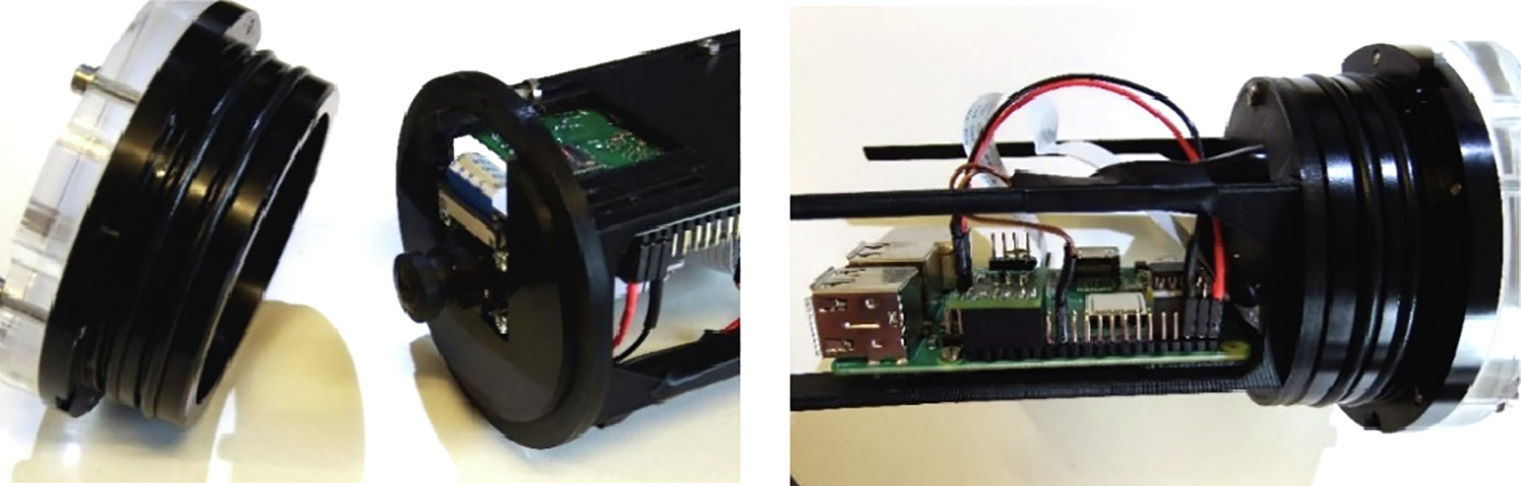
Attachment of the IR-server/camera to the flange.

The camera is mounted to the front gap with four flat head tapping screws 2.2 mm × 6 mm with the cable connector to the bottom. The Raspberry Pi is attached with four M2 × 12 mm screws (each a 3 mm spacers between the PCB and the mount) and hexagon nuts to the four slot holes on the mount. A CSI-cable connects the camera to the Raspberry Pi computer. It is inserted with the blue mark to the front at the cameras PCB and with the blue mark to the back of the Raspberry Pi (Fig. 9).

**Fig. 10:**
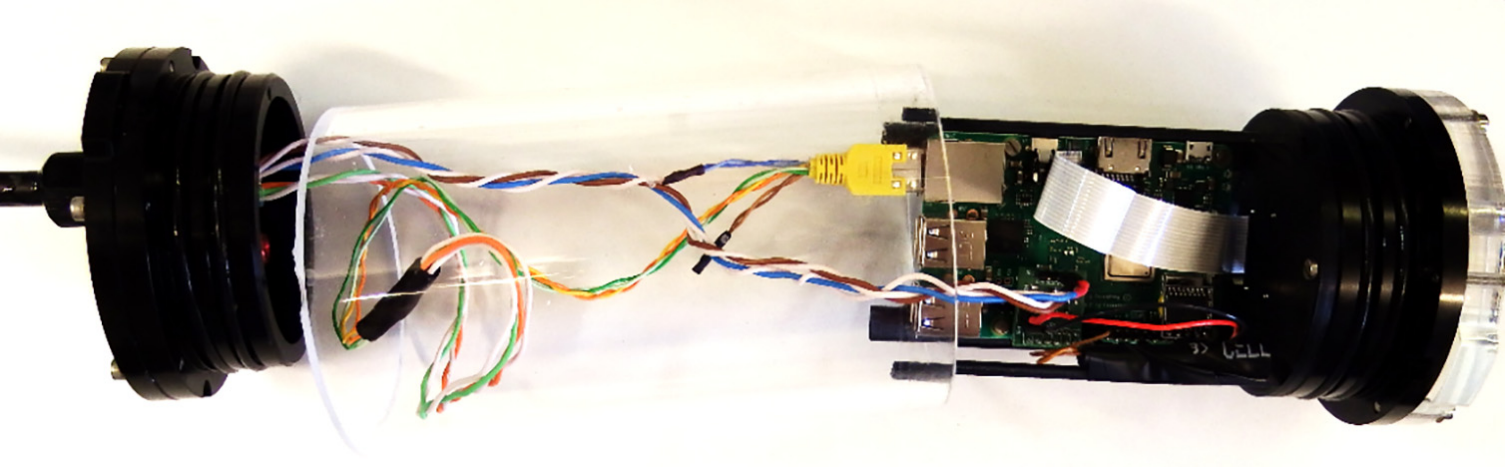
Cable assembly for IR server/camera.

The PCB mount is fixed with four M3 × 8 mm screws to the M3 threads of the front flange. The external power has to be connected to the connector J2 at the PCB ‘IR-server’. Finally, the electronics will be mounted inside the acrylic tube (enclosure) with the electrical connections tailored at the backside plate (Table 9). The underwater sealing procedure of the through-hole connectors is described at [18]. The underwater cable types and cable configuration depends on the requirements of the application. As power and ethernet cable for the server/camera, we use underwater ethernet cable like [19]. After sealing, the power and data lines are assembled according to Table 9. A simple connection of the Ethernet port of the Rasperry Pi can be made by cutting a standard patch cable, dismantle the sheath and solder the four data lines (Tx+,TX-,Rx + and Rx-) to the corresponding wires of the underwater cable. The power is tranferred according to 10/100BASE-TX with PoE pinout. The four wires are soldered to a two pin socket (2x 24VDC; 2x GND) (see Fig. 10).

**Table 9:** Server/camera: PoE-cable configuration; the input voltage range for 24VDC is 12-36VDC.

Pin/wire	Function	colour	Pin/wire	function	Colour
1	TX+	orange/white	5	24VDCb	blue/white
2	TX-	orange	6	RX-	Green
3	RX+	green/white	7	GNDa	brown/white
4	24VDCa	blue	8	GNDb	Brown

**Fig. 11:**
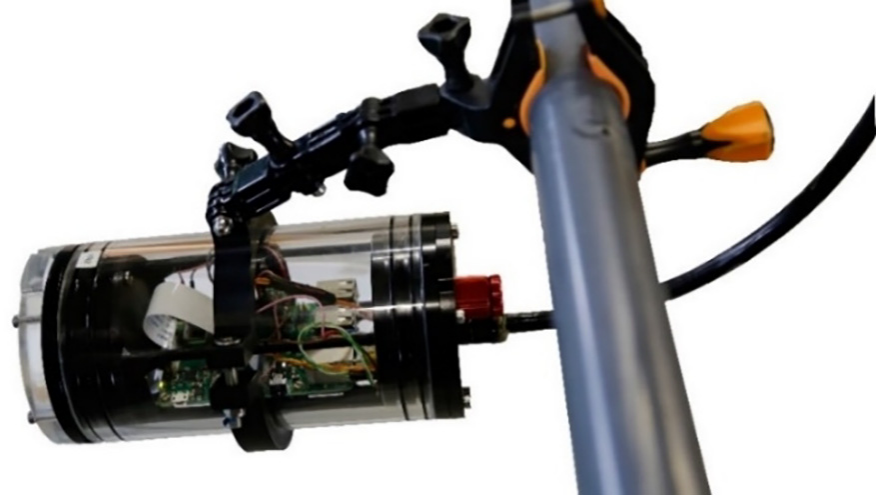
Assembled server/camera, attached to a frame using standard GoPro-clamps.

On the surface side, we use 9-pin WEIPU SP21 connectors to connect the underwater ethernet cable from the server/camera to the top side power and ethernet device. For easy installation we designed a 3D-printable mounting device that fits to all GoPro compatible mounts. A more detailed description with the mechanical drawings is given in chapter 3.2, Fig. 11 shows an assembled server/camera.

In summary the major assembly steps are:

1. Solder all parts to the PCBs.
2. Assemble the PCBs ‘IR-server’ and RTC to the RapsberyPi GPIO jumper.
3. Connect PCB ‘IR-server’ J4 power out to RaspberryPi GPIO jumper pins #2 (+5V) and #6 (GND) by a 2-wire line.
4. Insert the SD-card with the *iso*-image in the SD-card holder of the Raspberry PI.
5. Assemble the camera to the PCB mount.
6. Assemble the Raspberry Pi PCB to the PCB mount.
7. Connect the camera and the Raspberry Pi with a CSI-cable.
8. Assemble the PCB mount to the front flange of the enclosure.
9. Tailor the electrical connection at the backside plate to your needs and connect power in to J2 (pin #3 and #4) at the PCB ‘IR-server’ and the four ethernet wires to the RJ45 connector of the Raspberry Pi.

### 3.4 IR lamp

#### 3.4.1 Enclosure lamp

The housing for the IR lamp is the same as for the server/camera except, that the original tube is sectioned in four quarters of 74 mm length each. For a set of four lamps you need parts according to Table 10.

**Table 10:** Bill of material for the underwater housing (4 pieces).

Item	No.	Description	Manufacturer Code	Price/€
1	1	Cast Acrylic Tube – 11.75″, 298 mm (3″)	WTE3-P-TUBE-12-R1-RP	46.00
2	8	O-Ring Flange (3“	WTE3-M-FLANGE-SEAL-R2-RP	192.00
3	4	Aluminium End Cap (3″ Series)	WTE3-M-END-CAP-R1-RP	40.00
4	4	Clear Acrylic End Cap (3″ Series)	WTE3-P-END-CAP-R1-RP	40.00
5	4	Enclosure Vent and Plug	VENT-ASM-R1-RP	32.00
6	4	M10 Cable Penetrator for 8 mm Cable	PENETRATOR-M-BOLT-8MM-10-25-R2-RP	20.00
Total				370.00
Total for one lamp				92.50

A distributor list can be found in Table 3.

For sealing the cable penetrator we use potting compound (Table 8, Item15). The 100 g package can be used for appr. 35 cable penetrators, when all are sealed with one compound mix (within ½ an hour).

#### 3.4.2 PCB IR lamp

**Fig. 12:**
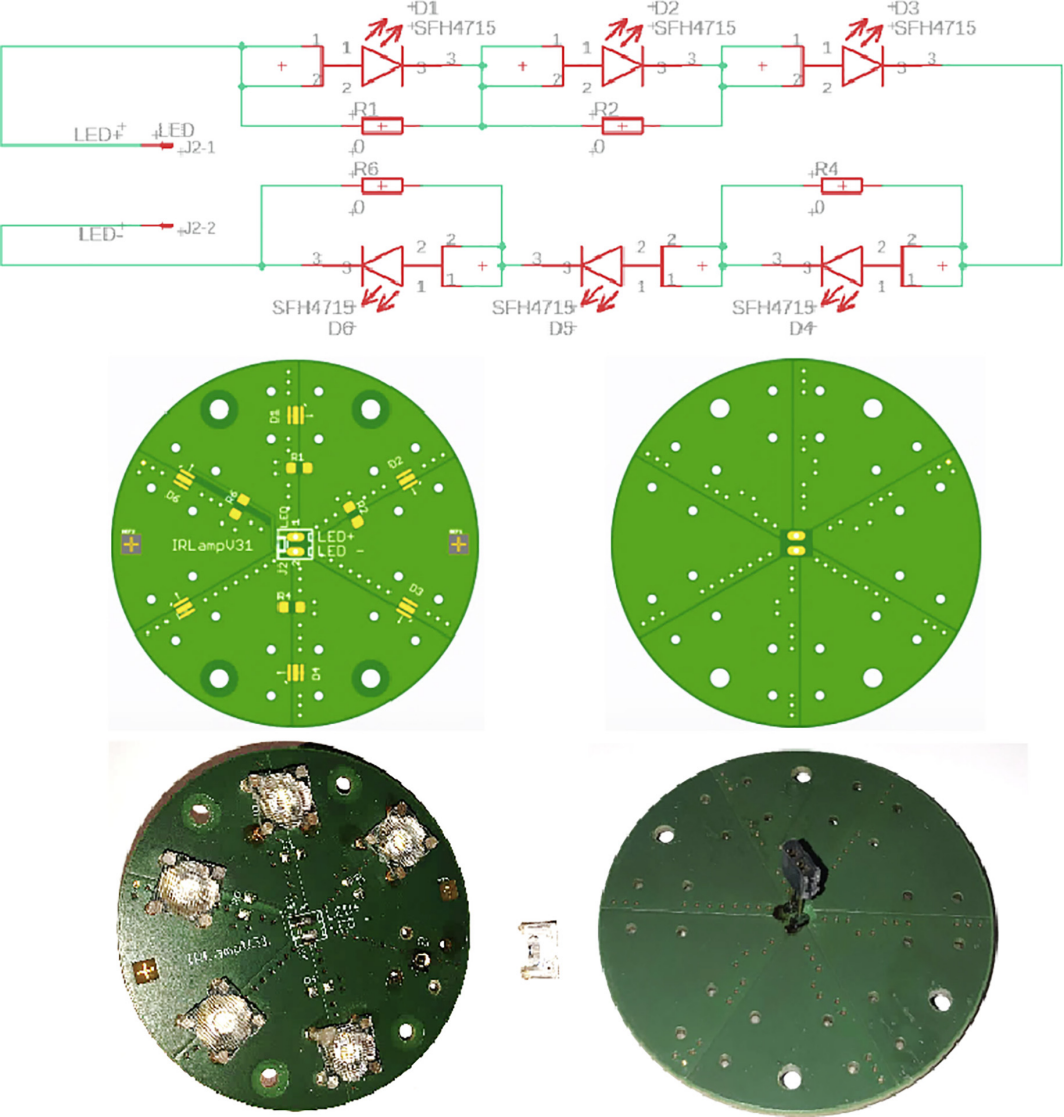
PCB IR-lampV31: Top: Circuit diagram; Mid: PCB layout in top and bottom view; Bottom assembled PCB with optional lens assembly (left), long pin-socket at bottom side (right).

**Fig. 13:**
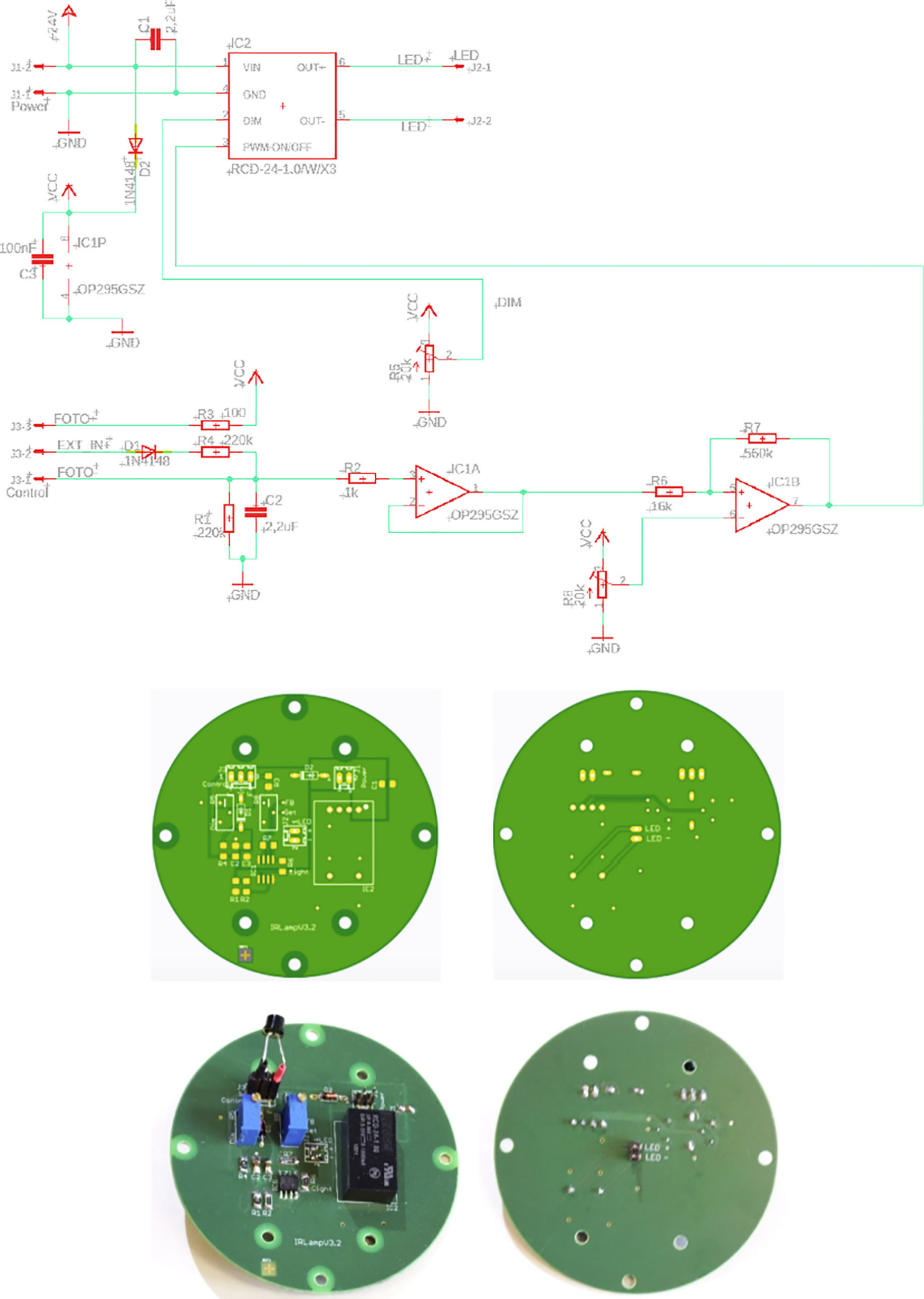
PCB ‘IR-lampV32′: Top: Circuit diagram; Mid: top and bottom view; Bottom: assembled PCB top and bottom view.

Two PCBs are required for each IR lamp: ‘IR-lampV31′ with six LEDs and ‘IR-lampV32′ for the LED driver. The schematics and PCBs are shown in Fig. 12 and Fig. 13.

The bill of material is given in Table 11 for four IR lamps. The six LEDs at each PCB ‘IR-lampV31′ are reflow soldered, while all other parts (including the SMD parts) can be also soldered manually. Both PCBs have four through holes to mount the PCBs to the M3-threads in the aluminium cooling mount. The PCB ‘IR-lampV32′ has 4 additional holes to mount it to the M3 threads in the flange of the underwater enclosure. When sufficient ambient light is available, e.g. during the day in shallow water, the IR illumination is not required. Therefore, there are two control options for the lamp:

1. Control by a light sensor
2. Control by the IR server/camera control signal

**Table 11:** Bill of material for the two PCBs of IR-Lamp (4 pieces each).

Item	No.	Description	Distributor / Code	Manufacturer / Code	Price/€
1	8	Capacitor SMD-3216 2,2uF/100 V	de.rs-online.com 136–4335	AVX SMD MLCC X7R 12061C225KAT2A	2.40
2	4	Recom LED-Treiber IC, 31 W, PCB 6-Pin	de.rs-online.com 668–9870	Recom RCD-24–1.0	44.48
3	8	OpAmp OP295GSZ SOIC8	de.rs-online.com 523–0284	AnalogDevices OP295GSZ	33.28
4	8	Diode 1 N4148	de.rs-online.com 739–0290	Schaltdiode 1N4148TA, 100 V 400 mA DO-35 2-Pin	0.88
5	8	Resistor SMD-1206 220kOhm 5% 0.5 W	de.rs-online.com 807–1173	TE-Connectivity CRGH1206J220K	0.24
6	4	Resistor SMD-1206 100Ohm 5% 0.5 W	de.rs-online.com 807–1104	TE-Connectivity CRGH1206J100R	0.12
7	4	Resistor SMD-1206 15kOhm 5% 0.5 W	de.rs-online.com 807–1132	TE-Connectivity CRGH1206J15K	0.12
8	4	Resistor SMD-1206 560kOhm 5% 0.5 W	de.rs-online.com 807–1255	TE-Connectivity CRGH1206J560K	0.12
9	20/36	Pin socket 1 × 2 2.54 mm	de.rs-online.com 549–0026	E-TEC 36-pin-socket BL1-036-G-700–01	1.87
10	8/20	Pin socket 1 × 2 2.54 mm long	de.rs-online.com 217–609	HARWIN 20-pin-socket D01B99520-42	0.83
11	28/36	Pin header 1 × 4 2.54 mm	de.rs-online.com 360–6342	Molex C-Grid III 20pin 90210–0780	1.55
12	4	Trimmpoti 3296Y 10kOhm 10% 1/2W	de.rs-online.com 522–0079	Bourns 25 Gang THT 3296Y-1-103LF	9.24
13	24	Osram Oslon Black LED ± 45° SFH4715S	de.rs-online.com 758–7646	OSRAM SFH4715S, 3 Pin	76.08
14	4	Phototransistor VTT9812FH	www.digikey.de VTT9812FH-ND	Excelitas Technologies	4.08
15*	24	OSRAM lens 10 mm med. spot frosted, 30°	www.lumitronix.com 60,387	Carclo SKU 60,387	26.88
15a*	24	OSRAM lens 10 mm plain tight, 20°	www.lumitronix.com 60,386	Carclo SKU 60,386	26.88
16	48/500	Slotted screw M3 × 8 mm	online-schrauben.de DIN84-4.8-M3X8	Steel galvanized DIN84-M3x8	0.96
17	16/100	Spacer round, Ø 6 × 3 mm, 3.2 mm drill	de.rs-online.com 102–6110	Richco 469.09.03	1.91
18	6/100	Potting compound 100 g	de.rs-online.com 199–1418	RS PRO Epoxid 2 × 50 g	0.54
19	4/12	PCB IRlamp_V31	www.aisler.net	PCB for playground 12 pcs	19.40
20	4/12	PCB IRlamp_V32	www.aisler.net	PCB for playground 12 pcs	25.88
21	1	Aluminium rod diameter 65, 63.5 or 60 mm, length 70 mm			
Total					250.86
Total for 1 piece					62.71

* Optional: depending on the application, different type of LED lenses can be used.

In our standard applications, we control the lamps by ambient light sensors (phototransistor VTT9812FH, Table 11, item 14). For a control by a light sensor, the phototransistors pins are soldered to a 3-pin socket with the anode at pin #1 and the cathode at pin #3, pin #2 is not connected. This 3-pin socket is connected to J2 at the PCB ‘IR lampV32′ (Fig. 13, bottom left). The setup for the control by the camera/server is described in chapter 3.4.5. The bill of material for the IR-lamps is given in Table 11.

#### 3.4.3 Aluminium cooling mount

**Fig. 14:**
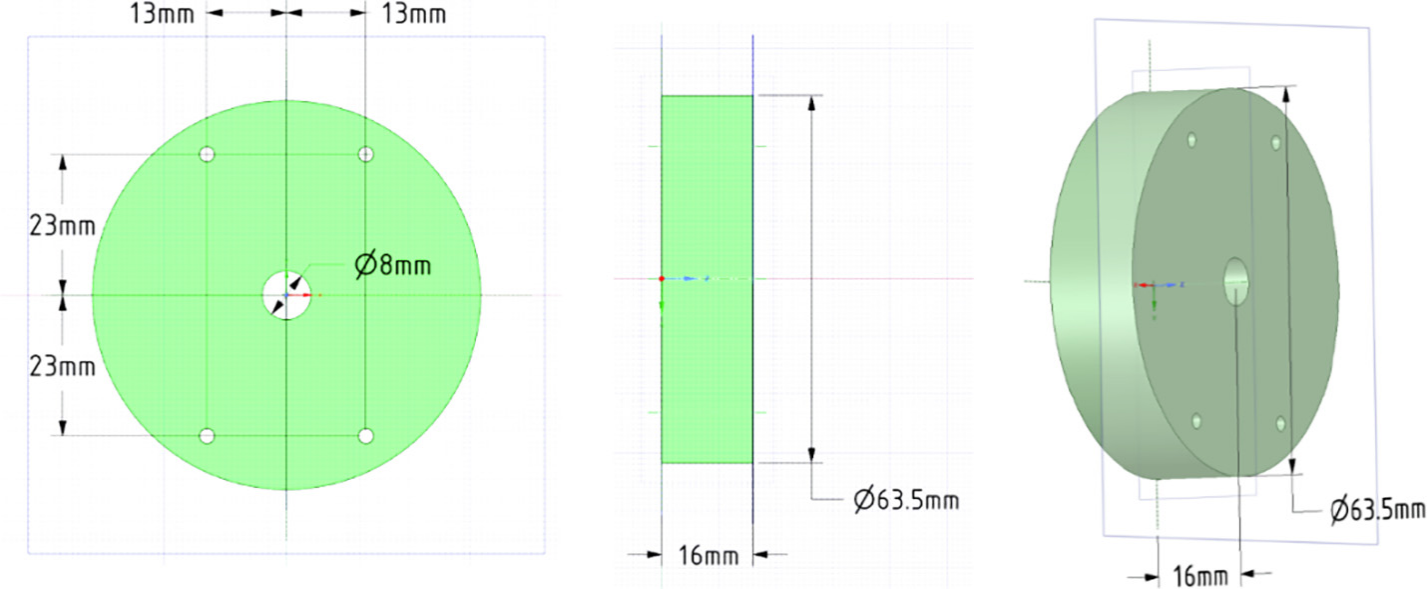
Drawing of the aluminium cooling mount for the two PCBs ‘IR-lampV31’ and ‘IR-lampV32’.

The two PCBs ‘IR-lampV31′ and ‘IR-lampV32′ are finally mounted to the aluminium cylinder that drains the heat from the LEDs to the aluminium flange and further to the surrounding water. The aluminium mount should have a diameter of 63.5 mm to fit best into the flange. We turned a 65 mm aluminium rod down to 63.5 mm and sawed 16 mm thick pieces from it, alternatively aluminium rods with diameters down to 60 mm can be used. The drilling were done by handcraft. There are four through holes with M3 threads to mount the PCB boards on the two sides of the cooling mount (Fig. 14).

#### 3.4.4 Assembly

**Fig. 15:**
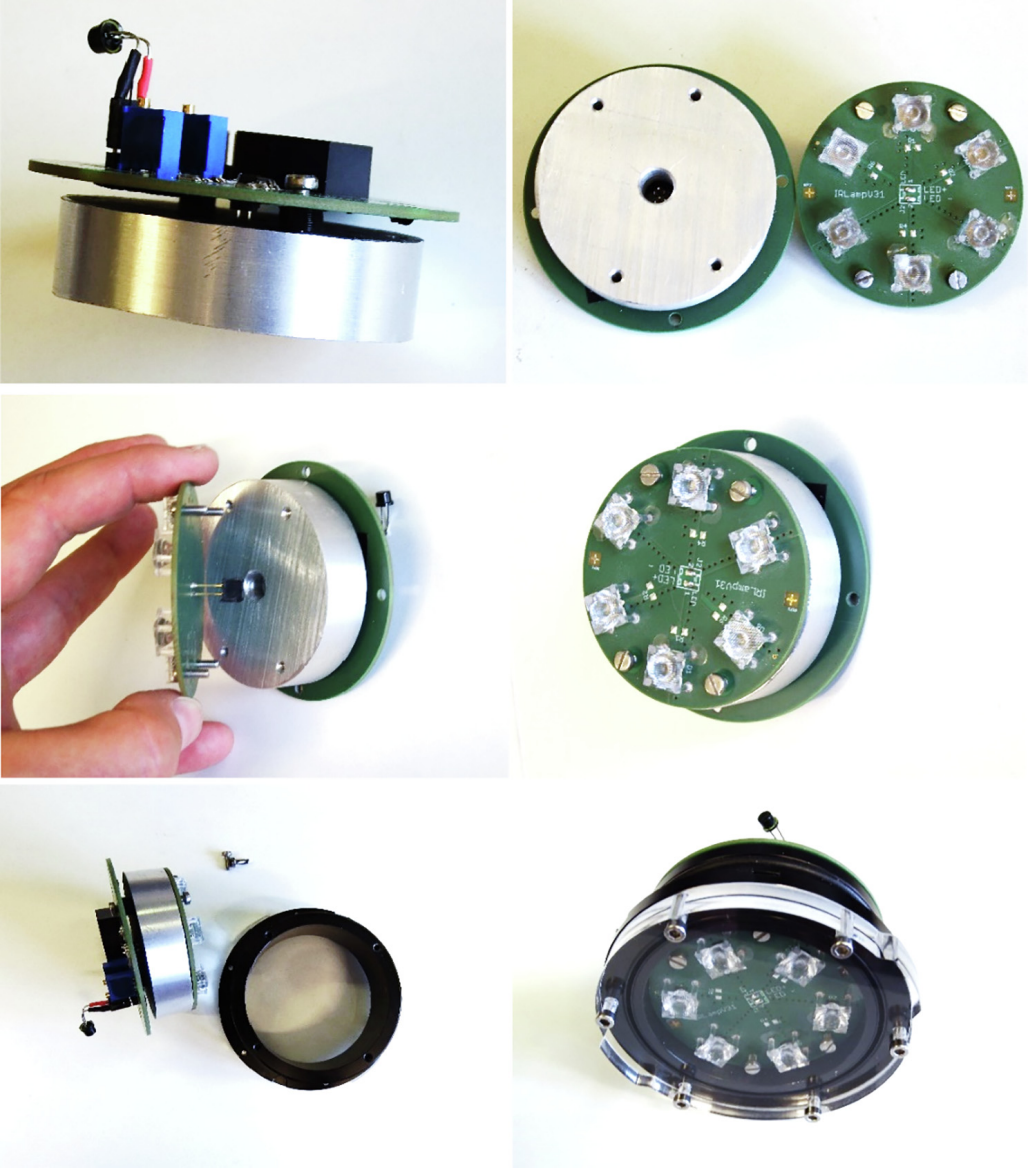
Assembly of the aluminium mount and the PCBs to the flange of the enclosure.

Fig. 15 shows the assembly of the PCB boards with the cooling aluminium mount from the top left to the bottom right. First, the PCB ‘IR-lampV32′ is mounted to the aluminium mount with four M3 × 8 mm screws and 3 mm spacers. Then the long 2-pole-pin socket of the PCB ‘IR-lampV31′ connected to the standard 2-pole pin header at the mounted PCB ‘IR-lampV32′ which is inside the 8 mm hole at the center of the aluminium mount. It is important to observe the correct polarity: LED + at ‘IR-lampV32′ has to be connected to LED + at ‘IR-lampV31′. The PCB ‘IR-lampV31′ is fixed directly to the aluminium mount using four M3 × 8 mm screws. To improve heat flow, thermal conductance paste between the bottom side of the PCB and the aluminium mount can be used. Finally, the block with the PCB boards and the aluminium mount is inserted into the flange and fixed with four M3 × 8 mm screws. Thermal conductance paste between the lateral area of the aluminium and the flange would increase heat flow even further.

**Fig. 16:**
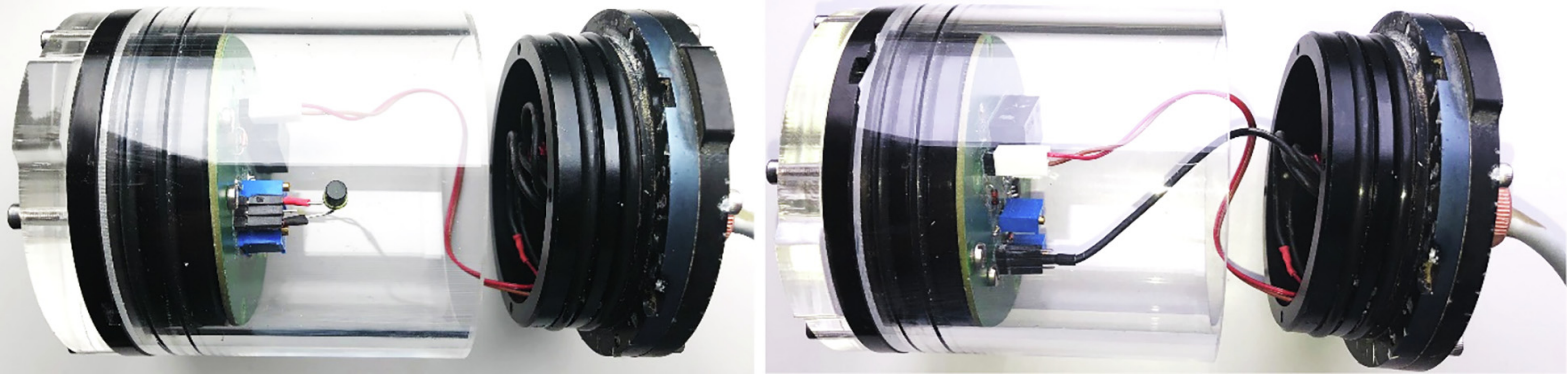
Cable assembly for IR lamp (left: ambient light control, right: Server/camera control).

The backside plate of the enclosure with its the electrical connections can be tailored to specific needs. The underwater sealing procedure of the through-hole connectors is described at [18]. Underwater cable types and cable configuration depend on the requirements of the application. In our application, we use 3-wire water resistant DIN VDE 0276 NYY-J 3x1,5mm^2^ cable and 3-pin WEIPU SP21 connectors to connect the lamps to the top side surface unit. After sealing, the power and data lines are assembled according to Table 12. The two power wires are soldered to a 2-pin socket, which is connected to J1 of the PCB ‘IR-lampV32′ with respect to the correct polarity. There are two options to control the lamps: by an ambient light sensor (standard) and remotely from the server/camera (optional) (see Fig. 16).

**Table 12:** IR-lamp: cable configuration (*for server/camera controlled lamp, see chapter 3.4.5).

Pin/wire	Function	colour	connect to
1	+24VDC	brown	IR-lampV32, J1, pin 1
2	Control	yellow/green	IR-lampV32, J2, pin 2*
3	GND	blue	IR-lampV32, J1, pin 2

**Fig. 17:**
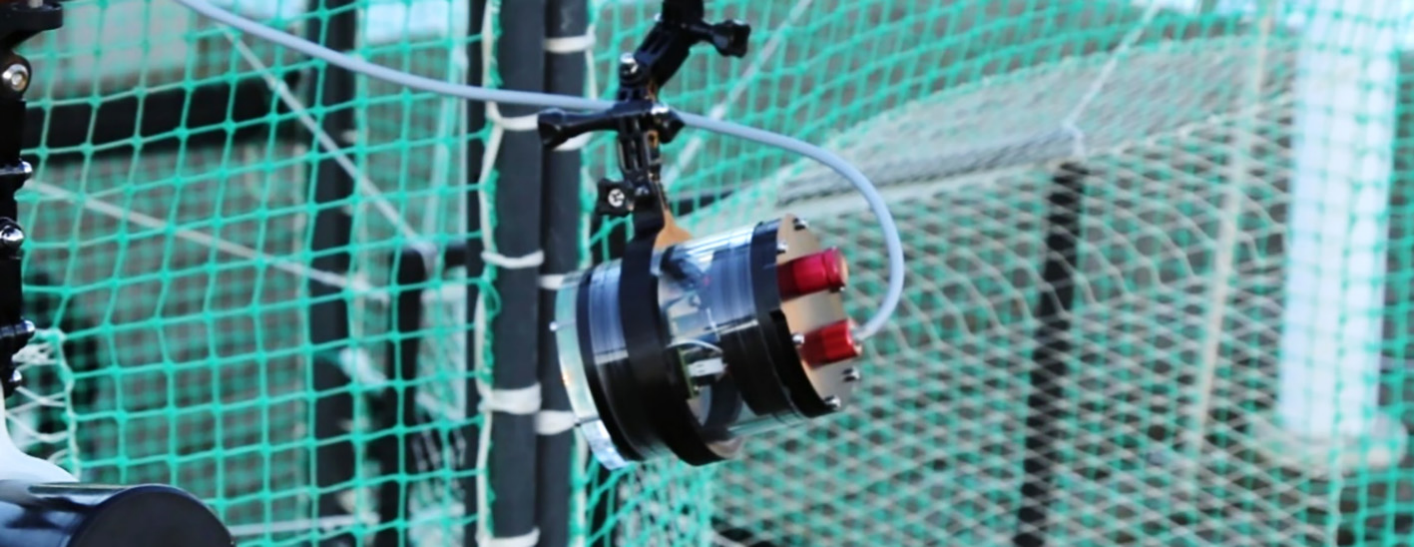
Assembled IR lamp with 3D-printed mount attached to a frame using standard GoPro-clamps.

For easy installation we designed a 3D-printable mount for the IR lamp that fits to GoPro compatible clamps. A more detailed description with the mechanical drawings is given in chapter 3.2. Fig. 17 shows an assembled IR-lamp mounted to a frame.

Major assembly steps are:

1 Soldering all parts to the PCBs ‘IR-lampV31′ and ‘IR-lampV32′.
2 Glueing optical lenses to the PCB ‘IR-lampV31′ (optional).
3 Assembling the PCB ‘IR-lampV32′ to the aluminium mount with spacers.
4 Assembling the PCB ‘IR-lampV31′ to the aluminium mount with respect to the polarity of LED+ and LED- connectors, heat conducting paste can be used.
5 Assembling the mount with its devices to the front flange of the enclosure.
6 Tailor the the electrical connection at the backside plate to your needs and connect the power to J1 at the PCBs ‘IR-lampV32’.

#### 3.4.5 PCB IR lamp under server/camera control

In our standard applications we control the lamps by ambient light sensors. For a remote control by server/cameras the cable setup is different and another cable type is needed. It either has two more wires for the lamp control, e.g. a underwater data cable with shielded 5 × twisted pairs (Table 13, Item 1) or one twisted pair with a greater cross-sectional area for power supply (Table 13, Item 2). It this case, the power supply is connected via the thicker twisted pair and the remaining twisted pair can be used for the two control lines.

**Table 13:** Cable types for server/camera remote operation of the lamps.

Item	No.	Description	Distributor / Code	Manufacturer / Code	Price/€
1	100 m	Helukabel 21037 Datenkabel LiYCY 5 × 2 × 0.25 mm^2^ Grau	www.conrad.de 1931447 – 62	AVX SMD MLCC X7R 12061C225KAT2A	144.04
2	1 m	SubConn PUR Cable P3TSP22#/1TSP18#	www.bornhoeft.de P3TSP22#/1TSP18#	SubConn PUR Cable P3TSP22#/1TSP18#	–

**Fig. 18:**
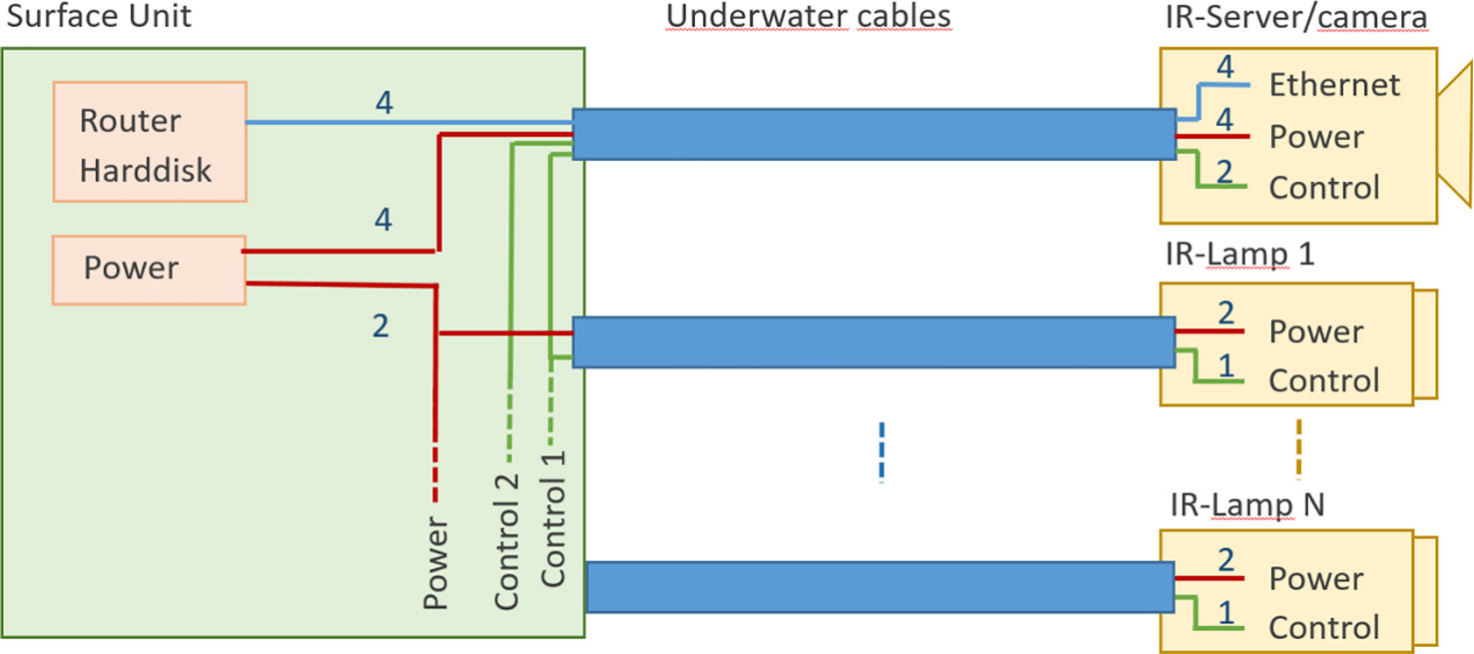
Wiring diagram for the remote lamp control in a surface unit.

For the remote control setup, the four twisted pairs for Ethernet are connected as described in Table 9. The two wires of the remaining twisted cable pair are connected to the lamp control outputs at J2 on PCB ‘IRserverV11′. Each of these wires can control a group up to six lamps. Therefore, the power-in-wires and the lamp control output wires are soldered to a 4-pin socket ordered as shown for JP2 in Table 7. This 4 pin-socket is connected to J2 on PCB ‘IRServerV11′ in the correct orientation. And, as in the standard setup, the 2-pin header JP4 is connected via a 2-wire line with pin-sockets to the power pins of the Raspberry Pi (GPIO jumper pins #2 (+5V) and #6 (GND)). Fig. 18 shows a wiring diagram.

In the surface unit, the control wires from the IR server/camera cable are connected to the control wire of cable to the lamps they should control. Two groups of lamps (‘Lamp control 1′ or ‘Lamp control 2) can be controlled by one server/camera. They are controlled by the GPIO Pins GPIO13 and GPIO21. It can be done by simple bash scripts, which can be accessed in the webservers scheduler or other LinuxOS scheduler (e.g. crontab).

For the remote control setup also the IR lamp wiring is modified. At first, the ambient light sensor is removed. The control signal wire (Table 12) from the underwater cable is soldered to a 3-pin socket at pin #2, while pin #1 and pin #3 are not connected. This 3-pin socket is connected to J2 at the PCB ‘IR- lampV32′ (Fig. 16, right). As in standard configuration, the two power wires are soldered to a 2-pin socket, which is connected to J1 (see Fig. 17, Fig. 18).

## 4 Software

**Fig. 19:**
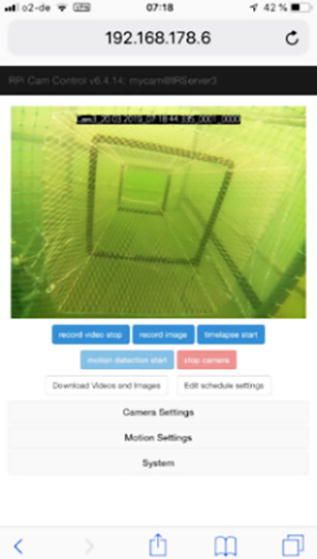
Screenshot of RPi-Cam-Control-Webfrontend on a mobile device.

The server/camera uses a Linux Debian OS without graphical XServer. The open source software RPi-Cam-Control [10], [11], provide a webserver which gives access to settings for camera and motion detection, as well as to basic system settings, like restart, power down or user defined functions (Fig. 19). Additionally, a live video stream and a comfortable scheduler are available, that can also start user defined scripts. A full documentation can be found at [12]. All software is packed as .iso image.

### 4.1 Setup the SD card image and access the server

The software for the server/camera is pre installed on the image file, that can be written to the SD-card using command “*dd”* under a LinuxOS or using the software Rufus (https://rufus.ie) under WinOS.

This SD-card has to be inserted into the Raspberry Pi before powering the server/camera. Two network connections are predefined, which can be modified after the first connection:

1. Ethernet connection: Static IP Adress: 192.168.178.6 Gateway 192.168.178.1
2. Wifi Connection: SSID: NKServer, Password: 3790 0606 0721 2004 9114 with DHCP enabled

The predefined connections were configured with the network command line interface “*nmcli”.* Both make it easy to install the cameras immediately on any FritzBox either directly wired or via Wifi, when the Wifi connection of the router is setup as predefined in the server/camera above. For a login the following credentials are valid:

**Hostname:** IRServer3 **User:** pi **Password:** irserver **Static IP:** 192.168.178.6

The server/camera is remote accessible via three protocols at its IP address:

1. via HTTP through the webserver interface,
2. via SSH connection to the Linux OS or
3. via SCP for file or video transfer from the camera to the remote system.

## 5 Use case with remote access via surface unit

In our specific use case, we wanted to improve fish pots for cod (*Gadus morhua,* L. 1758), especially the design of the pot entrance. Therefore, we needed a video surveillance system to observe cod behaviour in relation to different entrance designs – during day and night for several months.

The requirements were:

• observation at day and night
• minimum observation range: 2 m
• video data storage for several months
• fast swappable data disk
• motion detection
• remotely accessible
• webserver with live stream

**Fig. 20:**
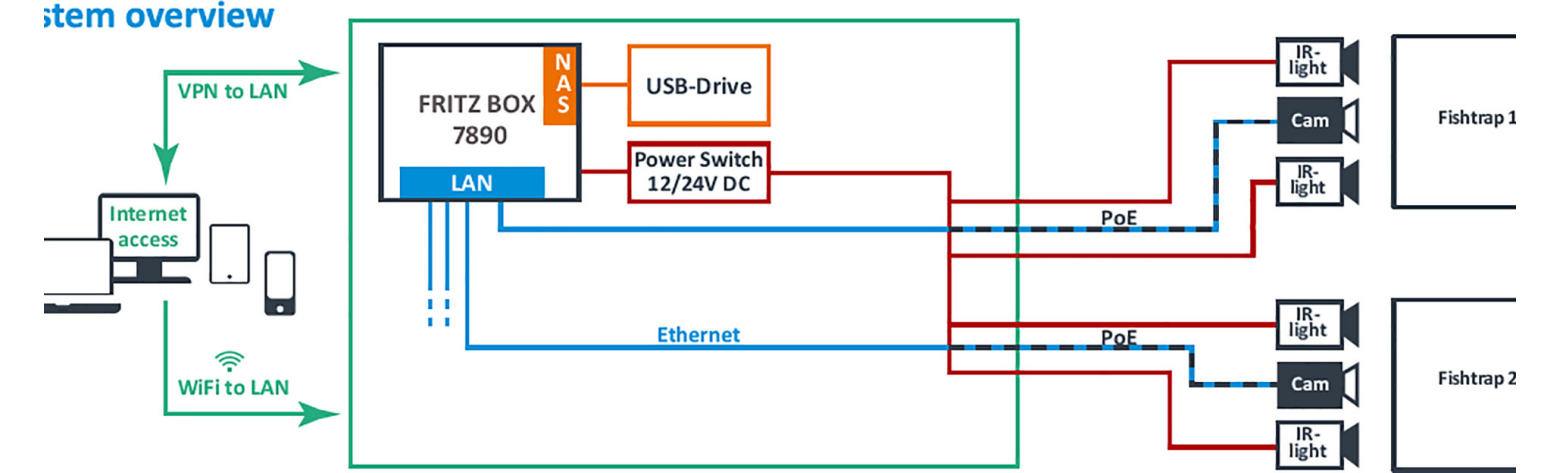
System overview of the remotely accessible system in the field.

In this use case the fish traps are located in shallow water (below 10 m depth) but we use the system also for applications in deeper waters (see specifications). A combination of two iFO systems is installed in the field (each with one camera and two IR-lamps). Both connected to an LTE router (FritzBox6890) with a swappable network attached storage (NAS) hard disk (Fig. 20).

**Fig. 21:**
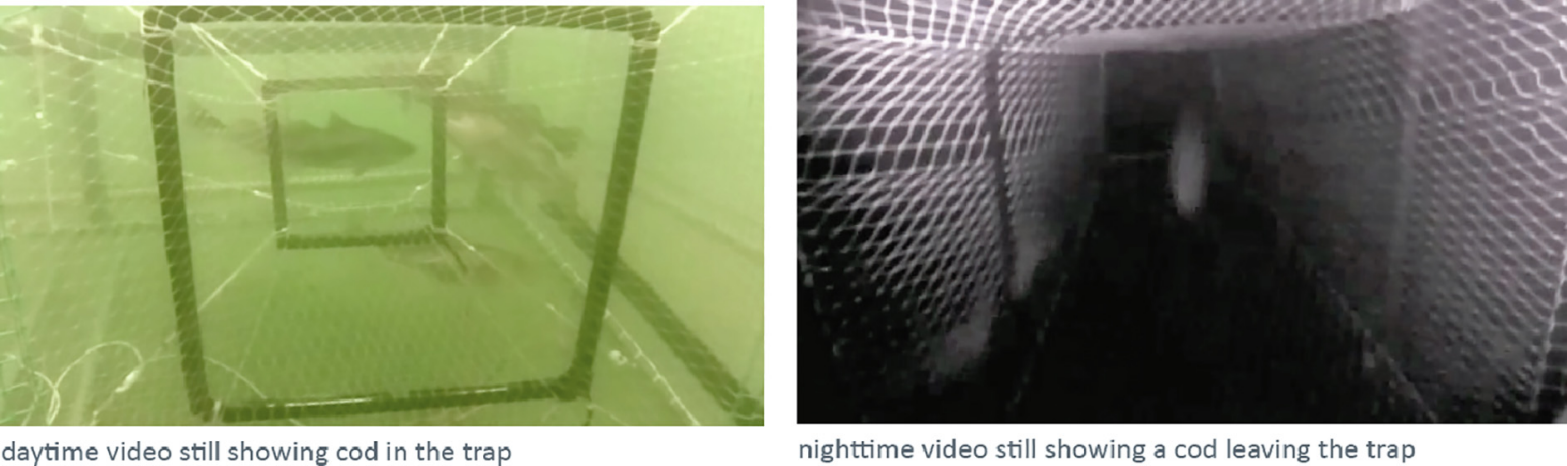
Daytime and nighttime video stills at the entrance of fish pots.

**Fig. 22:**
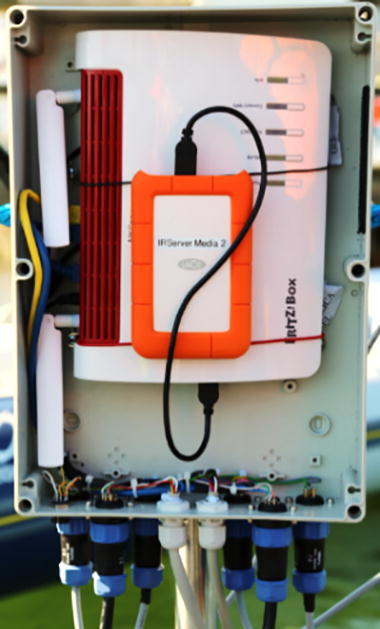
Surface unit of system in the field with LTE-router (FritzBox, NAS, plugs and wires; lid opened).

This allows continuous video data storage for several weeks and delivers full remote access via VPN and LTE to the underwater iFO systems. They are remotely accessible for live video streams, setup and administration, for video file downloads and maintenance (see Fig. 21, Fig. 22).

## 6 Summary and outlook

In this document, we present iFO, an open source low-budget underwater infrared video observation system with full documentation and sources to easily reproduce this. We briefly outlined one typical field of application, but we also use this system for other observation tasks, e.g. in fish tanks and off-shore. Of course, the system can be used in other environments and purposes as well: e.g. in harsh environments with the current underwater enclosures or in other environments with adapted ones. As a next step, we will derive a practical method from this work to estimate the underwater range of vision for infrared camera and light systems in general.

## Declaration of Competing Interest

The authors declare that they have no known competing financial interests or personal relationships that could have appeared to influence the work reported in this paper.
